# Supplementary material for: Are carbon emissions trading and green financial instruments synergistic? -Comprehensive quantitative research based on content analysis
Source: PLoS One. 2024 Mar 7;19(3):e0298601. doi: 10.1371/journal.pone.0298601 (PMC10919601; doi:10.1371/journal.pone.0298601)
Supplement: S1 Data — (ZIP) [file pone.0298601.s001.zip › Data/地级市Z_F工作报告.zip!/╣π╢1⁄2/╜¡├┼╩╨/└┤╫╘╣1⁄2╓┌║┼í╛┬φ┐╦╩2╛▌═°í┐_2018.docx]

各位代表：

　　我代表江门市人民政府向大会作工作报告，请予审议，并请政协各位委员和列席人员提出意见。

2017年工作回顾

　　过去一年，市政府坚持以习近平新时代中国特色社会主义思想为指导，在省委、省政府和市委的正确领导下，在市人大及其常委会的监督和市政协的支持下，围绕“兴业惠民、治吏简政”，扎实推进稳增长、促改革、调结构、惠民生、防风险、保稳定各项工作。全市地区生产总值2690.25亿元，增长8.1%；一般公共预算收入222.35亿元，增长10.98%；居民人均可支配收入26600元，增长9%；“放管服”改革走在全国前列，成功创建国家森林城市，全面完成了市十五届人大一次会议确定的各项目标任务。

　　一、实体经济持续壮大

　　产业平台扩容提质。大广海湾经济区作为重大合作平台写入《深化粤港澳合作推进大湾区建设框架协议》。完成粤澳（江门）产业合作示范区、珠西新材料集聚区、深江产业园、台山工业新城拓展区、开平翠山湖科技产业园拓展区等五大万亩园区土规调整和部分园区控规审定工作，启动6250亩土地平整工作。省产业园实现在三区四市全覆盖，连续6年获省级产业园考评优秀等次。传统产业不断优化升级，完成工业技改投资269.6亿元，增幅居全省前列。

　　先进制造业加快发展。出台“招商引资激励政策十二条”、“实体经济十条”，扶持力度是近年来最大的。引进超亿元产业项目109个、投资额835亿元，与正威集团签署投资产业项目战略合作协议，东风新能源商用车、电机产业城、优美科新能源材料、海目星激光科技、任达集团、华津精密金属、海亮二期等一批投资超10亿元重大项目落户。无限极、大长江、江粉磁材3家制造业企业产值超百亿元。珠江西岸先进装备制造产业带建设指标完成进度位居全省前列，装备制造业投资和增加值分别占全市工业投资和规上工业增加值39.6%和29.5%。

　　服务业势头良好。金融生态持续优化，两家企业成功在上交所挂牌上市，实现我市企业在上交所上市“零”的突破，目前全市在境内外上市企业达到20家。江门企业共协议吸收澳门金融机构资金67亿元人民币，拓展了融资渠道，降低了融资成本。市属交通建设和城市建设两大集团公司揭牌成立，江门农商银行组建取得实质性进展。举办第三届世界广府人恳亲大会暨2017中国侨都（江门）华人嘉年华，全国第9个、省首个联合国世界旅游组织可持续发展观测点落户江门，赤坎古镇华侨文化旅游项目进入实施阶段。商贸会展物流业加快发展，珠西国际会展中心投入使用，举办了第十六届广东教育装备展览会暨第二届江门台湾教育装备展览会等一批展会，普洛斯江门（鹤山）物流园开园，世界500强美国安博物流项目签约。全市接待游客5724.4万人次，旅游总收入达492.5亿元，分别增长13.7%和20.1%；完成外贸进出口总额1385.2亿元，增长9.8%；社会消费品零售总额1279.6亿元，增长10.4%。

　　现代农业稳步发展。推进农业供给侧结构性改革，新增省名特优新农产品36个、省农业类名牌产品11个、“三品一标”农产品10个、区域公共品牌核心企业6家和市级以上重点农业龙头企业12家，培育农业新型研发机构5家。恩平市成为首批国家农业可持续发展试验示范区创建单位，新会陈皮产业园成功进入国家现代农业产业园创建行列，台山中国农业公园通过创建验收。建成22.4万亩和新建20.17万亩高标准农田。荣获全省耕地保护考核综合一等奖。列入第二批国家农产品质量安全市创建试点单位，在全省率先建设农业投入品溯源信息平台，同步推进创建省食品安全示范城市，是全省唯一一个同步开展“双安双创”的城市。顺利完成第三次全国农业普查主体工作。农村综合改革不断深化，农村土地承包经营权确权登记颁证率达94.71%，走在全省前列；新会区国家农村集体产权制度改革试点、鹤山市国家级扶持村级集体经济发展试点等工作扎实开展。加强“三防”标准化建设，有效防御“天鸽”、“帕卡”双台风袭击，灾后复产工作顺利开展。

　　二、改革创新步伐加快

　　“去降补”成效显著。完成国有“僵尸企业”出清重组工作，商品房库存消化周期控制在合理区间，政府债务等重点领域风险防控工作扎实有效。实施“民营经济十二条”，为企业减负98.9亿元。“补短板”项目建设进展顺利。市重点项目完成投资634.9亿元，为年度投资计划的107.8%，带动全市固定资产投资完成1774.8亿元、增长16.9%。其中，交通大会战投资161.6亿元，增长20.7%。广中江高速二期、江门大道五邑路以北辅道、迎宾西路主线、胜利南路延长线、连海路一期、礼睦路扩建等21个交通项目建成通车，珠西综合交通枢纽江门站、开春高速、佛开高速南段扩建、开阳高速扩建、江门大道南（西线）等42个交通项目动工建设，中开高速、高恩高速加快建设。改造国、省道65.44公里，东部三区一市建成83个公交候车亭。江顺大桥获中国“鲁班奖”。

　　“放管服”大胆先行。在全国率先实施企业“十五证合一”和个体户“五证整合”改革，实现商事主体全覆盖，新登记市场主体6.4万户，创历年新高，全市市场主体总量突破30万户。在全省首批开通粤港澳银政直通车服务试点，实现对港澳商事登记离岸受理及远程办理。在珠三角地级市率先实施“证照分离”改革。积极推进清单制、承诺制、委托制改革，在全省地级市率先实施食品经营行政许可审批“申请人承诺制”试点改革；在全省率先开展县级跨部门“双随机一公开”联合抽查；率先实现“双随机一公开”监管“两个全覆盖”；率先实行工业产品生产许可证“先证后核”；率先推行投资项目承诺制改革试点，政府投资项目在全市范围内试行，企业投资项目在“1+6”园区试点推行。分两批下放或委托各市（区）行使市一级经济管理权限219项，向蓬江、江海两区下放土地收储权限。建立了市招商引资和工业园区开发建设两个联席会议制度，实施招商引资项目代办制。江门海关出口和进口通关效率分别在全国排第2和第8。蓬江区法人“一门式”改革获评“中国十大民生决策”奖项。

　　创新发展成绩突出。扎实推进珠三角（江门）国家自主创新示范区建设，高新技术企业数量达741家，省级工程技术研究中心达238家，均比上年翻了一番；规模以上和主营业务收入5亿元以上工业企业研发机构覆盖率分别达到43%和100%，达到和超过省定目标。“小微双创”示范工作扎实推进。科技企业孵化器达21家，众创空间达24家，均实现倍增。累计培育2078家科技型小微企业。江门高新区在全国综合排名上升了13位，获评“国家知识产权强县工程示范区”。台山市通过国家知识产权试点城市考核验收。拥有国家知识产权优势企业1家、实现“零”的突破，省级知识产权优势企业达26家。全市高新技术产品出口同比增长38.2%。实施“人才强市十四条”，新建院士工作站4个，目前全市已增加到7个；全国博士后创新（江门）示范中心建设扎实推进，引进院士1人、博士后15人、博士87人、硕士758人；高级职称专业技术人才达1.5万人，高技能人才连续6年每年增加万人以上，总数达12.6万人；江门“人才岛”首期项目开工建设。五邑大学列入广东省高水平理工科大学创建单位。成功获批创建全国质量强市示范城市，制定修订国家标准、行业标准、省地方标准11项。新增商标注册7000多件，创历年新高。

　　三、城乡面貌持续改善

　　环境整治力度空前。全面落实中央环境保护督察整改工作，170件交办信访举报案件全部办结。高规格成立江门市环境保护委员会，加强市环保监察执法力量，强化蓬江区、江海区的环保管理体制。在全省率先推行市县镇村四级河长制，天沙河、杜阮河、麻园河和会城河黑臭水体整治初见成效，龙溪河和紫水河消除重度黑臭；主城区西江沿岸烟囱基本拆除，淘汰高污染燃料锅炉349台，淘汰黄标车8394辆，超额完成省下达的任务；关停取缔“散乱污”企业618家、提升整治296家、加强监管263家；城镇生活污水处理率达到92.56%。国家海洋督察整改工作进展顺利。

　　城市建设扎实推进。加快编制《江门市城市总体规划（2017—2035）》，优化城市空间布局。完成《银湖湾滨海新城概念规划》编制和启动区的划定，加快推进江门大道南（西线）、银洲湖高速等基础设施和首批旅游项目的规划建设，推动城市沿江向海发展。启动江门市综合交通一体化规划修编。制定城市品质提升计划，完成华夏幸福产业新城启动区、礼东片区、江会路片区、万亩园区启动区、滨海新城启动区局部区域的控规覆盖，加快滨江新区、科创新城、枢纽新城、珠西物流产业新城等城市新区和产业集聚区的开发建设。

　　人居环境日益优化。顺利通过全国文明城市测评复查，3镇村3单位1学校获全国精神文明建设表彰，鹤山市获评省文明城市，台山市、恩平市分别列入全国、省文明城市提名城市。蓬江区棠下镇入选“全国特色小镇”。投入8.72亿元开展农村人居生态环境综合整治，累计完成自然村综合整治4104条，占全市20户以上自然村总数的46.65%。全市农村生活垃圾有效处理率达99.4%，在全省首创镇村生活污水设施建设“整县打包、整市推进”PPP实施模式。投入12.43亿元开展新一轮农村电网改造升级，全面完成192条中心村电网改造任务。完成碳汇林工程1.49万亩，建成生态景观林带90.5公里、乡村绿化美化点137个，新增森林公园14处。新会区崖门镇、台山市川岛镇、恩平市那吉镇和大田镇被认定为“广东省森林小镇”。镇海湾红树林湿地公园获批为国家湿地公园试点，建成5个镇街公园，新建改建154个村居公园，新建慢行系统和城乡绿廊145公里。深茂铁路江门至茂名段建设全球首例高铁拱形全封闭声屏障，保护小鸟天堂景区生态。

　　四、民生保障水平提升

　　持续加大民生投入。全市各级财政投入民生227.8亿元，增长15.3%，着力解决人民群众关心的热点难点问题，增进民生福祉。十件民生实事顺利完成，荣获“中国全面小康特别贡献城市”奖，人民群众获得感、幸福感、安全感明显增强。

　　社会事业不断进步。17家义务教育“三二一”工程的学校已有5家建成并投入使用，新增优质学位9720个。16个紧密型“医联体”试点分院已有12个基本达标或完工，对符合条件的困难群众医疗费用实行医疗保障兜底，100%提供全额资助。全市城镇新增就业4.82万人，城镇失业人员再就业3.28万人。全市城乡居民基本养老保险、基本医疗保险覆盖率分别达99.97%、98.92%。2家养老服务机构获颁“全国敬老文明号”。新建29家残疾人社区康园中心。江门职业技术学院接受省级示范校工作验收；设立广东江门中医药职业学院并已招生；启动广东江门幼儿师范高等专科学校创建工作，新校区开工建设。创建首个省级侨乡文化生态保护实验区，台山市海丝史迹考古遗址公园成为广东首个列入国家考古遗址公园立项名单的项目。举办2017年南粤古驿道定向大赛（台山海口埠站）、省“文化和自然遗产日”系列活动。

　　社会建设力度加大。“六项民生改革”成效明显，没有一个学生因贫失学和一个家庭因学致贫，户籍人口家庭医生签约率达50.7%，全面推进居民身份证异地办理、无户口人员落户。村委会年集体可支配收入全部达到18万元以上，5000名重点帮扶对象实现脱贫，重点帮扶对象100%实现“两不愁、三保障”。六项底线民生保障水平进一步提升，新增解决14.1万人农村饮水问题。超额完成省下达的棚户区新开工和公租房分配任务，提前一年全面完成农村危房改造任务。建立复退军人服务体系，提高义务兵优待标准。认真解决群众信访诉求，圆满完成党的十九大等一系列重大节点时段维稳安保工作，恩平市如期摘除“制毒重点关注地区”帽子，全市刑事治安警情比上年下降15%，刑事立案下降22.9%，社会治安持续向好。各类生产安全事故死亡人数持续下降，事故起数、受伤人数、经济损失实现两位数下降。

　　此外，全面启动对口广西壮族自治区崇左市扶贫协作工作；与黑龙江省七台河市建立对口合作关系。外事、港澳、对台、民族、宗教、双拥、人防、打私、统计、妇女儿童、新闻、出版、档案、地方志、气象、红十字会、应急等工作进一步加强。

　　五、政府建设不断加强

　　加强市政府党组建设，制定实施《关于贯彻落实〈中共江门市委关于加强党员干部教育监督管理构建良好政治生态的工作意见〉的意见》《关于贯彻落实党的十九大精神推进政府作风建设的工作意见》。落实中央八项规定精神，市本级财政拨款行政事业单位三公经费支出下降8.2%。抓紧政府系统党风廉政建设，严格落实“一岗双责”，加强审计工作，坚决查处违纪违法案件。

　　自觉接受人大和政协的监督，办理人大代表建议和政协提案满意率均达100%。“七五”普法规划全面实施，市政府常务会议平均每季度学法一次，全市制定2部地方性法规、1部政府规章，完成对现行规范性文件的清理工作，行政机关负责人出庭应诉率提高，市本级和7个市（区）、72个镇（街）获评省级法治创建先进单位。基本实现辖区公共资源交易流程（事项）电子化。政府决策的民主化、科学化、法治化水平进一步提高。

　　各位代表，过去一年是新一届政府的开局之年，全市经济社会发展所取得的显著成绩，是我们紧紧依靠市委的领导，在市人大、市政协的大力支持和港澳台同胞、海外侨胞的关心帮助下，全市人民、海内外投资者建设者以及驻江门军警部队共同努力的结果！在此，向所有参与、支持和关心江门发展的各界人士，表示崇高的敬意和衷心的感谢！

　　我们也清醒看到，江门的经济社会发展还存在着不少短板，进入了新时代，发展不平衡不充分的问题尤为突出：经济规模不大、发展质量不高，中心城区辐射带动能力弱，和珠三角核心区域相比，我市东部经济实力不强和东西部发展差距大的问题同时并存；产业结构调整力度不大，自主创新能力不强，新兴产业和现代服务业发展滞后；全市城市基础设施投入少，管理水平低，“脏乱差”问题比较突出；大气污染防治、黑臭水体治理形势依然严峻；针对困难群众和特殊群体的公共服务供给水平需要进一步提升；一些单位和干部不敢担当、服务意识不强、办法不多、效率不高，“跟不上、慢半拍”，等等。对此，我们必须高度重视，采取有力的措施予以解决。

2018年工作安排

　　2018年是贯彻落实党的十九大精神的开局之年，是改革开放40周年，是决胜全面建成小康社会、实施“十三五”规划承上启下的关键一年。今年市政府工作的总体要求是：以习近平新时代中国特色社会主义思想为指导，全面贯彻落实党的十九大和中央经济工作会议、中央农村工作会议精神，落实省委十二届二次和三次全会、市委十三届五次和六次全会精神，统筹推进“五位一体”总体布局和协调推进“四个全面”战略布局，按照“三个定位、两个率先”和“四个坚持、三个支撑、两个走在前列”的要求，坚持新发展理念，把握稳中求进总基调，围绕“兴业惠民、治吏简政”，坚持工业立市，深化供给侧结构性改革，构建全面开放新格局，稳增长、促改革、调结构、惠民生、防风险、保稳定，以高质量发展破解发展不平衡不充分的问题，打好防范化解重大风险、精准脱贫、污染防治三大攻坚战，为全面建成小康社会奠定坚实基础，确保新时代有新气象、新作为、新进步。

　　今年全市经济社会发展主要预期目标是：地区生产总值增长8%，固定资产投资增长12%，社会消费品零售总额增长10%，进出口总额增长3%；地方一般公共预算收入增长9%；研发经费支出占地区生产总值比重达2.1%；居民消费价格涨幅控制在3%以内；居民人均可支配收入增长略高于经济增长；城镇新增就业4.5万人以上，城镇登记失业率控制在3.2%以内；城镇化率达65.6%；节能减排约束性指标和大气、水环境质量确保完成省下达的目标任务。为此，要着力抓好以下工作：

　　一、积极主动融入大湾区建设

　　深入推进交通大会战。加快粤港澳大湾区西翼枢纽门户城市建设，年度安排大会战项目105项，投资165.7亿元。打通承东启西的高快速路网。加快中开高速尤其是银洲湖大桥和双水至水步段建设，实现全市东西部快速连接，抓好高恩高速、开春高速、佛开高速南段扩建、开阳高速扩建等工程建设，启动银洲湖高速建设、江鹤高速扩建，开展珠海香洲至台山高速、珠海斗门至恩平高速、广台高速开平至台山段等前期工作。加快江门大道南、台开快速路建设，完成江门大道东甲至三江段、银鹭立交至小冈大桥段建设，启动三江至南门大桥段建设，推进滨江快线（江侨路—南山路）项目前期工作。新改建国、省道14.55公里，改造国、省道路面64.8公里。加快迈进轨道交通新时代。深茂铁路江门至茂名段上半年全线通车，珠西综合交通枢纽江门站年底基本建成站房主体工程，加快推进深茂铁路江门至深圳段、江肇高铁、广佛江珠城轨、广珠铁路客运改造、南沙港铁路、江门北站货场扩建等项目规划建设，优化全市轨道交通一体化规划。启动主城区轨道交通规划。完善多层次综合交通体系。完成《江门市综合交通一体化规划（2017—2035）》编制工作，形成全方位对接港珠澳大桥、深中通道、虎门二桥和珠三角干线机场，快速联通三区四市以及重大产业发展平台的大交通格局。充分发挥西江黄金水道的作用，加快高新区公共码头建设，完善港口集疏体系，积极推进台山市广海湾鱼塘港及开放口岸建设。完善通用机场规划布局，推进恩平冯如通用机场报批、台山通用机场选址等前期工作。

　　咬定招商引资不松劲。今年是“招商引资突破年”，要把招商引资作为加快发展实体经济的“牛鼻子”，紧抓不放，各市（区）、各部门务必满腔热情、不畏困难、不辞劳苦，主动走出去，用足用好优惠政策，推动精准招商、产业招商、敲门招商、以商引商，加快新项目落户建设，重点抓实超10亿元的重大优质工业项目落地，着力提高项目落地率、开工率、达产率、财税贡献率。扎实推进珠江西岸先进装备制造产业带建设，加快打造轨道交通、重卡和专用车、新材料新能源及装备、教育装备、大健康等产业集群，力争在新能源电池、激光装备、智能终端等新兴产业取得突破。

　　加快打造重大产业平台。加快推进粤澳（江门）产业合作示范区、珠西新材料集聚区、深江产业园、台山工业新城拓展区、开平翠山湖科技产业园拓展区等五大万亩园区建设，新增“三通一平”土地5000亩，提高园区基础设施建设水平，承接珠三角核心区产业投资溢出。加快全市产业发展布局规划，明确各类园区产业定位及准入条件，提高产业生态链建设水平。各市（区）要继续推进现有核心园区扩能增效，处置一批低效用地和厂房，提升园区节约集约用地水平。

　　推动产业优化升级。提升机电五金、电子电器、水暖卫浴、纺织服装、食品等传统产业发展水平，鼓励企业开展技术改造和增资扩产，完成技改投资增长20%以上，引导企业应用工业互联网，深化制造业与互联网融合以及大数据、人工智能应用推广。大力扶持龙头企业，鼓励企业兼并重组，抓好建筑业等优势产业和新业态依法纳入统计，推动外地企业到江门集群注册，发展总部经济，力争产值超百亿元企业新增1至2家。推动中车广东公司加快发展。加强外贸品牌和质量建设，培育外贸新业态新模式，促进外贸稳增长。加强口岸基础设施建设，推进国际贸易“单一窗口”服务，提升通关便利化水平，抓好大广海湾保税物流中心（B型）申报建设工作。加快现代物流、工业设计、专业会展等生产性服务业发展，办好摩托车博览会，推动实体零售创新转型发展，促进消费增长。以创建全国质量强市示范城市为抓手，促进全市产品质量、工程质量、服务质量和环境质量上新台阶。筹建国家轨道交通轻合金材料质量监督检验中心，加快国家级珠西综合质量检测平台建设。

　　统筹区域协同发展。深化大广海湾融入粤港澳大湾区发展的战略研究。推进东部一体、西部协同发展新格局。科学编制新一轮城市总体规划、城乡重点发展区域规划，全面开展我市第三次全国土地调查，推进“多规合一”和主体功能区精准落地，推动中心城区的规划一体化，着力破解发展不平衡不充分的问题。蓬江区要坚持新兴产业和城市经济双轮驱动，当好排头兵，建设全市的首善之区；江海区要发挥国家级高新区优势，当好全市创新发展的引领区；新会区要抓住珠西综合交通枢纽和区内三个万亩产业园区的建设机遇，争当全市做大总量、提高发展质量的主力军；台山市要抢抓港珠澳大桥即将通车、大广海湾上升为大湾区发展平台的机遇，积极谋划，全力争取大项目落户，在台开恩发展中争当“领头羊”；开平市要强化打造重大产业平台和经营城市理念，推进工业、旅游和城市品质全面提升；鹤山市要发挥对接广佛经济圈的桥头堡优势，做大工业和物流业，推进产城融合，推动经济社会发展进入江门“第一梯队”；恩平市要坚持绿色发展，加大园区、产业提质增效的力度，提高发展质量水平。高标准建设“四个新城”。滨江新区立足于打造滨水园林新城区，加快完善交通路网、城市综合管廊、公共配套等基础设施建设，打造总部经济基地；科创新城加快集聚创新资源，增强国家级高新区创新功能；枢纽新城以大项目建设为抓手，打造以江门站为核心的枢纽城市新格局；银湖湾滨海新城加快推进新城定位和发展思路研究、重点建设区控制性详细规划编制、用地和用海报批等前期工作，加大产业园区建设力度，加快交通等基础设施建设，加强岸线资源管控，启动一批旅游项目建设。加快各市（区）城镇化进程，推进江会路片区融合，加快城市扩容提质，促进江门中心城区功能的提升和拓展。搭建湾区合作新平台。充分发挥我市港澳同胞和华侨资源丰富的独特优势，着力在发展平台、交通设施、城市建设、科技金融、文化旅游、侨资侨智等方面与粤港澳大湾区建设进行全方位对接，深化江澳金融合作，推动江深合作向纵深发展。

　　大力引进培育人才。大力实施人才政策，建立人才数据库，搭建人才交流对接服务平台。高标准建设全国博士后创新（江门）示范中心。立足为大湾区聚集人才，今年“人才岛”启动总投资额超50亿元项目建设，加快环岛景观带升级改造、国际教育培训中心、人才公寓等项目建设。加强省市共建，全力支持五邑大学创建高水平理工科大学。服务好国家中微子实验基地的建设。筹建江门人力资源服务产业园，举办工匠讲堂，加快培养紧缺技能人才。完善人才服务保障体系，着力解决人才落户、住房、子女入学入托、出入境等突出问题。

　　擦亮侨乡文化品牌。推进侨务强市建设，办好第六届世界江门青年大会，深入推进中国（江门）“侨梦苑”华侨华人创新产业聚集区建设。加快推进省级侨乡文化生态保护实验区建设，推进以“白沙文化”为重点的优秀传统文化传承工作，开展原创文艺精品推广计划，深入挖掘咏春、蔡李佛等侨乡武术文化。加强周郡、甘化厂历史遗迹等文化资源的保护开发，提炼和传承红色文化基因。弘扬梁启超家族爱国报国家风，倡导文明好家风。继续推进海丝申遗，筹办海上丝绸之路文化遗产立法保护研讨会。完成市文化馆、美术馆改造，升级改造五邑图书馆、华侨博物馆，加快全市国家综合档案馆达标及市档案中心项目建设。举办2018中国（江门）侨乡文化产业博览会、2018·广东（江门）振兴传统工艺推介会，保护文化产业版权，促进文化产业发展。推进城乡文明建设，完善基层综合性文化服务中心，全市镇（街）文化站100%达到一级站以上，村（社区）60%达标，推动文化惠民“进学校、进军营、进社区、进农村、进企业”，举办300场以上的文化惠民活动，广泛开展群众文化艺术活动。

　　加快旅游强市建设。抓好中国国际特色旅游目的地创建，台山市和开平市加快创建国家全域旅游示范区。推动各市（区）积极创建A级旅游景区和“醉美江门100村”建设。开平市重点加快碉楼文化旅游区创建5A工作，整体提升自力村、马降龙村等景区质量。加快开平市赤坎古镇、新会区美丽湖湾、宋元崖门海战遗址、台山市滨海旅游、鹤山市珠三角乡村生态旅游目的地、恩平市地热地质生态旅游等龙头项目招商、建设。积极配合省滨海旅游公路的规划建设。统筹推动文化、体育、旅游协同发展。

　　二、大力推进深化改革和创新驱动发展

　　扎实推进供给侧结构性改革。加快交通、电力、水利、通信等领域的投入，完成基础设施投资420亿元以上。落实“去降补”任务。全面清查东部三区一市重点区域的化工、玻璃、皮革、印染、火电等重污染行业，结合中心城区环保、“三旧”改造和安全生产需要，实施政策引导，推进化工专区建设，加快产业整体搬迁，精准推动污染和危化企业加快退出中心城区。以江门大道沿线为突破口，加大“三旧”改造力度。鼓励各市（区）加强对存量土地、低效用地、低效厂房的盘活利用，确保2013—2017年间批准的建设用地供地率达到60%以上。支持工业企业提高土地容积率，鼓励零地增资扩产，建设高标准厂房，实行工业上楼。加快建立多主体供给、多渠道保障、租购并举的住房制度，促进房地产市场平稳健康发展。坚决打击违法违规金融活动，用好用足政府债券、PPP模式、政府购买服务政策，规范政府和社会资本合作，防范化解政府债务风险。落实电力体制改革政策，推动建立国华台电、台山核电与本地工业企业电力市场交易的协调机制，在能源、物流、用地等领域全面降低制造业企业成本，促进实体经济提质增量。积极推进第四次全国经济普查工作。

　　深入推进“放管服”改革。围绕“两无两藏”，深化清单制、承诺制、委托制改革，推动政府职能转变。进一步完善“1+3+N”开放型清单，深化行政许可、公共服务目录清单和实施清单标准化工作，推进全市各级行政服务办事大厅、网上办事大厅、部门审批服务平台与清单数据统一规范对接。加快投资项目在线审批监管平台建设，力争投资项目承诺制改革在全省乃至全国取得率先突破。加强各市（区）承接能力建设，把委托制改革落到实处。深入推进“证照分离”、“多证合一”等商事制度改革，压减审批事项和环节，拓宽住所申报制改革范围，多渠道推动商事登记离岸受理，全面推行“互联网+商事登记”模式。全面推进跨部门“双随机”联合抽查，推进全市涉企信息统一归集，实现“双随机一公开”监管常态化、制度化、规范化。

　　激发市场主体活力。打造公平市场环境，促进各类市场主体做大做强。发展壮大国资国企。加快市属交通建设和城市建设两大集团公司的实体化和资本化运营，积极推进市汽运集团、市碧源公司等一批国有企业上市。加快国有企业的市场化进程，创新优质资产注入模式，鼓励企业参与园区开发、土地营运、“三旧”改造、城市建设。提高国资监管水平，严控投资风险和债务风险，确保国有资本安全运行、保值增值。做优做强金融产业。加快市金控公司发展，积极参股银行、保险公司等金融机构，整合壮大地方性金融机构。3月底前新会农商银行和江门融和农商银行完成合并组建为江门农商银行；6月底前完成鹤山农信社改制为农商银行；年底前落实台山、开平两家农信社改制为农商银行相关申报筹建工作。研究筹建江门华侨银行。积极扶持民营经济。构建亲清新型政商关系，鼓励中小企业向“专精尖”提升，培育更多细分行业“单打冠军”、“隐形冠军”。加强金融对企业的支撑，大力支持企业上市，培育上市后备企业资源，引导中小企业利用多层次资本市场直接融资。支持行业协会（商会）发展。

　　加快珠三角（江门）国家自主创新示范区建设。全面落实创新驱动发展“八大举措”，启动国家创新型城市创建工作，推进江门珠西科技产业创新服务中心建设。加快高新区扩容提质，支持台山工业新城、开平翠山湖科技产业园申报省级高新区。增强企业自主创新能力，高新技术企业总数突破1000家，科技企业孵化器达30家，省市级新型研发机构达30家，省级工程技术研究中心达300家，省级以上创新平台（研发机构）超330家，规上工业企业设立研发机构比例超50%。

　　营造良好创新生态。深化与广东省科学院等高校和科研院所的产学研合作，促进科技成果的转移转化。加强培育国家知识产权示范城市工作，启动专利申请增量提质行动。完善科技金融服务体系，大力发展科技贷款、贷款贴息、融资担保业务，加大“邑科贷”、“研发贷”推广力度，推进知识产权质押融资和专利保险试点。深化“小微双创”，做好迎接国家检查验收工作，科技型小微企业总数超3000家，完善珠西智谷、珠西创城等“小微双创”核心基地建设。大力推动“个转企”、“小升规”，实现小微企业上规模250家。加快推进特色小镇建设，打造宜创宜业宜居宜游的新型创新创业平台。大力倡导崇尚创新、宽容失败的创新文化，激发全社会创新创业活力。

　　三、着力建设美丽江门

　　加快生态文明建设。启动国家生态文明建设示范市创建工作。坚持铁腕治污，深入推进环境执法百日整治行动，按期完成中央环保督察、省大气和水污染防治专项督查的整改任务。按照三年规划、主要目标任务2018年基本完成的要求，大力推进环保达标行动，坚决打赢大气、水、土壤污染防治攻坚战。改善空气质量。强化颗粒物和臭氧污染控制，协同减排挥发性有机污染物与氮氧化物，加快高污染燃料禁燃区建设，推进工地文明施工，加强城市扬尘、移动源污染防控精细化管理，进一步深化区域大气联防联控和重污染天气应对。整治水环境。全面严格落实河长制，加强饮用水源保护，完成新一轮镇村污水处理设施建设任务，城镇生活污水处理率达到95%，确保潭江牛湾断面稳定达到Ⅲ类水质，市区6条黑臭水体全面消灭黑臭现象。防治土壤污染。启动土壤污染状况详查，科学编制治理与修复实施方案，加强源头监管。保护海洋环境。坚持陆海统筹，全面完成国家海洋督察整改任务，出台养殖用海规划和海洋生态环境保护规划，落实海洋生态红线管控措施。巩固“创森”成果。持续深化森林城市建设，完成生态公益林扩面17万亩，优先将高速公路沿线一公里等重点区域、生态环境脆弱区域的林地区划为生态公益林。整治高速公路、国道沿线采石场，保护森林覆盖面貌。加强森林消防工作。积极推进各级各类公园建设，加快推进新会区小鸟天堂国家湿地公园、开平市孔雀湖国家湿地公园、镇海湾红树林国家湿地公园、蓬江区天沙湖湿地公园、江海区西江外滩生态湿地公园、台山市金星湖公园、开平市白云石森林公园、鹤山市昆仑山森林公园、恩平市响水龙潭森林公园等9个新公园建设，建成镇街公园5个，新建改建村居公园138个。

　　全面提升城市品质。以确保全国文明城市成功复牌为目标，今年投入215.2亿元开展城市品质提升“八大行动”，力争3—5年城市面貌、城市品质迈上新台阶。推进城市更新。大力开展“城市修补、生态修复”，更新改造老旧城区，活化利用历史街区和建筑，对老旧小区开展综合整治，加快老旧管线改造，重点解决“脏乱差”问题。完善城市给排水、燃气、通信、电力等基础设施，提高市政基础设施承载力。推进“厕所革命”，推动厕所建设标准化、设施现代化、管理规范化。开展交通治堵行动。进一步提升城市交通水平，完善城市快速路网、步行和自行车等慢行系统以及公共停车场建设，建立道路微循环系统。完善公交一体化发展，推进公交站场建设，优化公交线网。提升城市景观环境。围绕江边、河边、商业街区、旅游景区等开展城市美化亮化绿化工程，加强户外广告管理，实施临街建筑外立面整治，打造一批街道景观升级示范路。加强市容环境管理，完善卫生保洁“一把扫”机制和城乡生活垃圾统筹收运处理体系，推进生活垃圾分类减量。加强城市精细化管理。深化城市执法体制改革，大力整治“两违”，加强对噪声扰民行为的整治，提升城市治理效能和水平。加快布局发展高速宽带和5G网络，推进智慧城市、海绵城市建设。

　　加快实施乡村振兴战略。深入推进农业供给侧结构性改革，加快现代农业和美丽宜居乡村建设。坚持质量兴农、绿色兴农。大力培育名特优新农产品，新增市级以上重点农业龙头企业10家、农民专业合作示范社10家、家庭农场15家。完成国家农产品质量安全市创建验收，推进新会陈皮国家现代农业产业园建设，提升恩平市国家农业可持续发展试验示范区建设水平，加快建设广东省农产品加工示范区、珠西农产品交易流通中心和“江门现代种业长廊”，加快三产融合。落实粮食安全责任制。改善农村生产生活。不断提高城乡宜居水平，增强对农业转移人口的吸引力和承载力。抓好农村人居环境整治，深入推进名村示范村和生态村、镇创建。完成20.17万亩和启动13.18万亩高标准农田建设，完成垦造水田1.5万亩，加快海堤达标加固工程、中小河流治理工程、病险水闸除险加固工程、灌区改造工程、村村通自来水工程等项目建设。把农村公路建好管好护好营运好，实现全市行政村客运班车通达率达100%。推进新会区崖门渔港升级改造项目开工建设，完成台山市烽火角区域性避风锚地建设。推进农村综合改革。全面完成农村土地承包经营权确权登记颁证工作，完善承包地“三权”分置制度；基本完成鹤山市国家级扶持村级集体经济发展试点、新会区国家农村集体产权制度改革试点工作；加快江海区的区、街、村共同开发村级留用地新模式试点工作。

　　四、努力促进民生事业发展

　　优先发展教育事业。加快推进义务教育“三二一”工程学校建设，确保年内全部建成。推动学前教育优质普惠发展，建立健全城镇住宅小区配套幼儿园的管理机制，提升优质幼儿园比例，积极应对明年学前教育入园高峰的到来。推动义务教育均衡化、标准化、一体化发展，小学适龄儿童入学率达到99.99%，合理配置教育资源，引导品牌学校集团化。高中阶段毛入学率达到95%以上，提升高中办学整体水平。建设高素质教师队伍。继续改善贫困地区孩子的上学条件。办好优质民办学校，大力发展民办教育。加快江门幼儿师范高等专科学校创建及新校区建设，创建职业教育综合改革示范市、农村职业教育和成人教育示范县。

　　提高就业和社会保障水平。深化就业实名制，鼓励多渠道多形式就业，全市城镇失业人员再就业3万人。鼓励创业带动就业，新增3家创业孵化基地。完善收入分配制度，加强企业最低工资调控，大力构建和谐劳动关系。推进企业职工养老保险省级统筹，稳步实施医保城乡一体化改革，推进跨省异地就医联网结算。提升养老服务质量，实现每千名老人拥有养老床位36张以上，支持养老产业加快发展，民办及公建民营、公办民营养老机构占比达30%以上。重视老年教育。完成360户贫困重度残疾人家庭无障碍改造任务，确保全市有需求的残疾儿童和持证残疾人接受基本康复服务的比例达80%以上。加快慈善事业发展。加强复退军人服务体系建设。积极推进老旧城区住宅加装电梯工作。

　　大力建设健康江门。加快创建卫生强市，深化医药卫生体制综合改革，办强公办、引进民办，打造珠西医疗中心。继续推进“医联体”建设，完善分级诊疗、基层首诊和双向转诊制度，努力实现“大病不出县”。大力推进强基创优行动，全面开展县级公立医院、医疗急救体系标准化升级建设，完成197间村卫生站的规范化建设，健全家庭医生签约服务激励机制。完善中医药服务体系。加快推进医疗、医药、医保“三医联动”，深化医保支付方式改革。加强重大疾病防控，提升卫生应急能力水平。全面铺开健康城市、健康村镇试点建设工作。加强妇幼保健机构标准化建设，强化优生优育服务。加强食品药品监管，创建广东省食品安全示范城市，建成7条省级食品安全示范街，“明厨亮灶”覆盖率达到80%。积极申办第十六届省运会，加快市体校扩建和陈白沙体育广场等场地建设，深入推进全民健身与全民健康深度融合，打造全民健身品牌赛事。

　　坚决打赢脱贫攻坚战。落实市、县、镇三级结对挂钩扶贫工作机制，持续推进扶贫线与低保线“两线合一”改革试点，深化“百医牵百村”、“百企扶百村”、“千万义工助千户”等社会帮扶行动，全面完成建档立卡的5097户16659人贫困人口精准脱贫任务。完善基本公共服务均等化补助和资源管护激励型财政补贴政策，实现村委会年集体可支配收入全部达到20万元以上。治理扶贫领域腐败和作风问题。持续提高城乡最低生活保障、特困人员供养、孤儿供养、城乡医疗救助、困难残疾人生活补贴和重度残疾人护理补贴、城乡居民养老保险基础养老金等六项底线民生保障项目水平，实现低保标准城乡一体化，完善重特大疾病医疗救助制度。

　　打造平安江门。加快推进综治视联网和“雪亮工程”建设，实施智慧新警务，落实三年禁毒工程，不断完善社会治安防控体系，严厉打击和惩治黄赌毒黑拐骗、“盗抢”等违法犯罪活动。严格执行维稳综治及安全生产党政同责、一岗双责、齐抓共管、失职追责，排查整治重点领域安全隐患，深入推进城市风险点、危险源排查管控，坚决遏制较大以上事故发生。完善突发事件应急机制，提高应急管理能力。高标准完成市县镇三防系统标准化建设。建设“平安海洋”等气象保障工程。开展放心消费创建活动，优化消费环境。进一步完善“大调解”工作格局，健全社会矛盾纠纷预防化解机制，充分发挥社会组织作用，进一步深化志愿服务制度化建设，支持江海区建设全国农村社区治理实验区，完善城乡社区治理。完善社会稳定形势分析研判、重大决策社会稳定风险评估、矛盾纠纷批量政策性解决等机制，从源头上解决影响社会稳定的突出问题。

　　做好上述民生工作，必须加大民生投入，全市各级财政安排民生支出254亿元，增长12%。深入推进基本公共服务均等化综合改革，实施城乡居民增收行动计划和公共服务提升行动计划，抓好十件民生实事落实，补齐民生社会事业短板，让全市人民共享改革发展成果。进一步完善国防动员体系，继续做好外事、港澳、民族、宗教、对台、双拥、优抚、人防、打私、统计、地方志、妇女儿童、红十字会等工作。积极推进与广西壮族自治区崇左市四个县的“携手奔小康”帮扶行动，开展与黑龙江省七台河市的对口合作。

　　五、持续加强政府自身建设

　　推进依法治市。主动接受人大监督，认真执行市人大及其常委会各项决议决定，支持政协委员参政议政，提高人大代表议案、建议和政协提案办理水平。落实市人大常委会年度立法计划，认真组织起草并提交地方性法规草案，继续深入推进政府规章和政府规范性文件制定工作。严格规范公正文明执法，开展高标准法治创建活动，各市（区）、镇（街）创建率达100%，村（社区）创建率达90％以上。进一步完善市县镇村四级公共法律服务实体平台建设，统筹全市政府法律顾问资源，落实“谁执法谁普法”的普法责任制，开展全民普法工作。深化机构和行政体制改革，推进承担行政职能事业单位改革和生产经营类事业单位改革。推进财政预算管理改革。

　　推进“马上就办”。大力提倡“马上就办、真抓实干”的工作作风，克服“跟不上、慢半拍”的习惯，积极争取上级支持，加强上下联动，建立为市（区）解决突出问题的机制，推进下基层抓落实常态化，立说立行、紧抓快办、限时办结。实行招商引资项目行政审批代办制，提高投资项目审批速度。加快推进数字政府建设，全面深化“一门式、一网式”政府服务改革，实施不动产登记集成服务等改革。拓展“侨都之窗”自助服务终端机应用范围，开拓手机APP、政务微信等服务渠道，提升“12345”政府服务热线水平，力求事事有着落、件件有回音，为群众、企业提供便捷高效的政务服务。

　　推进阳光政务。全面推进政务公开，自觉接受社会和舆论监督。健全市、县两级财政支出进度通报制度，完善财政预算执行管理。推进第三方评估行政审批和政务服务效能，开展网上行政许可在线监管和实时评价工作，逐步推动评估结果运用。推进审计监督全覆盖。加强诚信政府建设和社会信用体系建设，完善守信激励失信惩戒制度，推进各市（区）、各系统、各部门信用记录的整合。加强公共资源交易活动的规范管理，深化公共资源交易“大数据”应用，推动监督方式向“数字化”转变。

　　推进廉政建设。坚持作风建设永远在路上，不断巩固和拓展落实中央八项规定精神的成果，深入查找和纠正“四风”突出问题特别是形式主义、官僚主义的新表现。加强党章党规党纪和法律法规教育，严格落实党内监督条例，强化不敢腐的震慑，扎牢不能腐的笼子，增强不想腐的自觉。严格落实各级政府、各部门党风廉政建设责任制，加强审计和巡察结果运用，持之以恒正风肃纪反腐，树立清正廉明的政府形象。

　　各位代表，做好2018年各项工作，任务艰巨，时不我待。让我们更加紧密团结在以习近平同志为核心的党中央周围，在省委、省政府和市委的坚强领导下，不忘初心、牢记使命，开拓进取、干在实处，为江门的加快发展而努力奋斗，推动党的十九大精神在侨乡大地落地生根、开花结果。
